# THE PANC 3 SCORE PREDICTING SEVERITY OF ACUTE PANCREATITIS

**DOI:** 10.1590/0102-6720201600010002

**Published:** 2016

**Authors:** Murilo Gamba BEDUSCHI, André Luiz Parizi MELLO, Bruno VON-MÜHLEN, Orli FRANZON

**Affiliations:** General Surgery Division, Hospital Regional de São José Dr. Homero de Miranda Gomes), São José, SC, Brazil

**Keywords:** Pancreatitis, Predictive value of tests, Pleural effusion, Intensive care

## Abstract

***Background*:**

About 20% of cases of acute pancreatitis progress to a severe form, leading to high mortality rates. Several studies suggested methods to identify patients that will progress more severely. However, most studies present problems when used on daily practice.

***Objective*:**

To assess the efficacy of the PANC 3 score to predict acute pancreatitis severity and its relation to clinical outcome.

***Methods*:**

Acute pancreatitis patients were assessed as to sex, age, body mass index (BMI), etiology of pancreatitis, intensive care need, length of stay, length of stay in intensive care unit and mortality. The PANC 3 score was determined within the first 24 hours after diagnosis and compared to acute pancreatitis grade of the Revised Atlanta classification.

***Results*:**

Out of 64 patients diagnosed with acute pancreatitis, 58 met the inclusion criteria. The PANC 3 score was positive in five cases (8.6%), pancreatitis progressed to a severe form in 10 cases (17.2%) and five patients (8.6%) died. Patients with a positive score and severe pancreatitis required intensive care more often, and stayed for a longer period in intensive care units. The PANC 3 score showed sensitivity of 50%, specificity of 100%, accuracy of 91.4%, positive predictive value of 100% and negative predictive value of 90.6% in prediction of severe acute pancreatitis.

***Conclusion*:**

The PANC 3 score is useful to assess acute pancreatitis because it is easy and quick to use, has high specificity, high accuracy and high predictive value in prediction of severe acute pancreatitis.

## Abstract (pt)

***Racional*:**

Cerca de 20% dos casos de pancreatite aguda evoluem de forma severa, acompanhados de alta mortalidade. Diversos estudos têm sugerido métodos para identificar pacientes que evoluirão com maior gravidade. Entretanto, a maioria apresenta problemas em sua utilização na prática diária.

***Objetivo*:**

Avaliar a eficácia do escore PANC 3 na predição da severidade da pancreatite aguda e sua relação com o desfecho clínico.

Pacientes com pancreatite aguda foram avaliados quanto ao sexo, idade, índice de massa corporal (IMC), etiologia da pancreatite, necessidade de cuidados intensivos, tempo de internação hospitalar, período necessário de cuidados intensivos e mortalidade. O escore PANC 3 foi determinado nas primeiras 24 h do diagnóstico e comparado ao grau de pancreatite aguda da classificação de Atlanta Revisada.

***Resultados*:**

Dos sessenta e quatro pacientes, cinquenta e oito preencheram os critérios necessários para inclusão no estudo. O escore PANC 3 foi positivo em cinco casos (8,6%), a pancreatite evoluiu de forma severa em 10 (17,2%) e 5 (8,6%) faleceram. Pacientes com escore positivo e pancreatite severa, necessitaram mais frequentemente de cuidados intensivos e, quando necessitaram, permaneceram por período maior nas unidades de cuidados intensivos. O escore PANC 3 demonstrou sensibilidade de 50%, especificidade de 100%, acurácia de 91,4%, valor preditivo positivo de 100% e valor preditivo negativo de 90,6% na predição de pancreatite aguda severa.

***Conclusão*:**

O escore PANC 3 é útil na abordagem da pancreatite aguda, por ser de fácil e rápida aplicação, apresentar alta especificidade, alta acurácia e alto valor preditivo na predição da pancreatite aguda severa.

## INTRODUCTION

Acute pancreatitis is a disease of miscellaneous etiology^3^^,^ 28. It is defined as acute pancreatic inflammation due to activation of digestive enzymes present in the interior of the gland, which affect the pancreas, adjacent tissues and other organs^9^^,^ 23.

Although in most of cases it presents a mild and limited course, the manifestation spectrum is broadly variable. Approximately 20% of patients have a severe form, with systemic complications, leading to high mortality^9^.

The management of acute pancreatitis is directly based on its severity^4^, for patients with severe disease will benefit from an intensive care since the onset of symptoms^5^^,^ 26. Hence the importance to estimate as early as possible how the disease will progress^9^.

Since the paper by Ranson, in 1974^19^, several studies suggested markers and scores to identify patients at risk of developing severe acute pancreatitis^16^^,^ 29. However, established tests present problems in daily practice use^5^^,^ 26. There is still no consensus on an ideal method, and the need of an objective way to predict acute pancreatitis severity remains^17^^,^ 27.

The advantages of the PANC 3 score are to employ widely available tests that are quickly performed and easy to measure, and to have high accuracy when predicting severe acute pancreatitis^5^.

The aim of the present study was to evaluate the efficacy of the PANC 3 score in predicting severity of acute pancreatitis and its relation with the clinical outcome of the disease.

## METHODS

The present study was approved by the Research Ethics Committee of the institution. All patients diagnosed from March 2013 to August 2014 as acute pancreatitis and seen by the General Surgery Division team, of the Hospital Regional de São José Dr. Homero de Miranda Gomes, São Jose, SC, Brazil, were evaluated.

The patients who agreed to participate in the research signed an informed consent form. However, if they could not be contacted directly by the researcher, their data were gathered from the medical charts. The collected data were kept confidential and stored by researchers, according to the ethical confidentiality standards.

The exclusion criteria were patients who chose not to participate in the study, those referred by other institutions and diagnosed as acute pancreatitis, with no properly collected data for inclusion in the study, and patients who were not duly followed-up.

The diagnosis of acute pancreatitis was made based on the presence of two out of the three following characteristics: 1) epigastric abdominal pain frequently radiating to the back, intense, persistent and of acute onset; 2) serum amylase and/or lipase levels three-fold higher than the upper normalcy limit; and 3) computed tomography with characteristic findings.

The severity of cases during hospital admission was assessed by the Revised Atlanta classification^4^.

The PANC 3 score was determined by measuring three variables obtained within the first 24 h after diagnosis of acute pancreatitis: 1) serum hematocrit; 2) body mass index (BMI); and 3) pleural effusion on the chest X-ray. The case was considered positive if serum hematocrit was >44 mg/dl, BMI>30 kg/m^2^, and pleural effusion was detected on the chest X-ray.

The demographic data collected to characterize the epidemiological profile of the study population were gender and age.

The cause of acute pancreatitis was determined by the history and imaging studies (ultrasound, computed tomography or magnetic resonance).

 Length of hospital stay, need of intensive care - defined as patient´s transfer to the Intensive Care or Stepdown Units -, length of stay in the intensive care unit, and mortality were also evaluated.

The data gathered were submitted to statistical analysis using the following tests: equality of two proportions, chi-square (χ²), Yates correlation (Y) and ANOVA, and expressed as means. The p-values less than 0.05 were considered significant.

## RESULTS

A total of 64 patients were diagnosed as acute pancreatitis; in that, 20 had prospective diagnosis (interviews) and 44 had retrospective diagnosis (data collected from charts). Six patients were excluded: one chose to not participate in the study; one was referred by another institution with incorrect collection of data; and four were not properly followed-up (one was transferred to another hospital and three were discharged upon request). Therefore, the final study population was composed of 58 patients, mostly women (63.8%), mean age of 48.5 years (range 20-86) and mean BMI of 29.9 kg/m^2^. The etiology of pancreatitis included biliary tract disease (84.5%), hypertriglyceridemia (5.2%), alcohol use (3.4%), neoplasms (3.4%) and idiopathic cases (3.4%). The PANC 3 score was positive in 8.6% of cases. Pancreatitis was mild in 75.9% of patients, moderately severe in 6.9% and severe in 17.2%. The mean length of hospital stay was 32.8 days, 19% of patients required admission to intensive care unit (ICU), and the mean length of stay at the ICU was 2.8 days. The mortality rate was 8.6% (Table 1).

**TABLE 1:** - Participant´s profile (n=58)

**Characteristics**		**p-value**
Gender		.003
Male	36.2% (21)	
Female	63.8% (37)	
Age (years)	48.5 (20 - 86)	
BMI (kg/m2)	29.9 (19.6 - 51.3)	
Etiology of acute pancreatitis		<.001
Biliary	84.5% (49)	
Non-biliary	15.5% (9)	
Hypertriglyceridemia	5.2% (3)	
Alcoholic	3.4% (2)	
Neoplastic	3.4% (2)	
Idiopathic	3.4% (2)	
PANC 3		<.001
Positive	8.6% (5)	
BMI (kg/m2)	34.2 (30.1 - 41.1)	
Hematocrit (mg/dL)	48.5 (45.4 - 55.2)	
Pleural effusion	100% (5)	
Negative	91.4% (53)	
BMI (kg/m2)	29.5 (19.6 - 51.3)	
Hematocrit (mg/dL)	39.6 (29.5 - 48.2)	
Pleural effusion	9.4% (5)	
Severity of acute pancreatitis		
Mild	75.9% (44)	
Moderately severe	6.9% (4)	<.001
Severe	17.2% (10)	<.001
Length of hospital stay (days)	32.8 (1 - 104)	
Required intensive care	19% (11)	
Length of stay (days)	2.8 (0 - 44)	
Mortality	8.6% (5)	<.001

BMI=body mass index

All patients with positive PANC 3 score had severe acute pancreatitis. However, half of patients with severe disease and the remaining had a negative score (Table 2).

**TABLE 2:** - Classification of patients according to the PANC 3 score and severity of acute pancreatitis as per the Revised Atlanta classification

**Criterion**	**Mild**	**Moderately Severe**	**Severe**
PANC3 +	-	-	5
PANC3 -	44	4	5

Comparing the PANC 3 score and clinical outcome, the positive score had no correlation with length of hospital stay and mortality. However, patients with positive score more often required intensive care and, in such cases, stayed longer at the ICU (Table 3).

**TABLE 3:** - Correlation between the PANC 3 score and clinical outcome

**PANC 3**	**Length of stay (days)**	**p-value**	**Intensive care required**	**Mortality % (n)**	**p-value**			
			**% (n)**	**p-value**	**days**	**p-value**		
Positive	30.0 (1 - 90)	0.798	80% (4)	0.002 (Y)	14.6 (0 - 44)	<0.001	20% (1)	0.343
Negative	33.1 (4 - 104)		13.2% (7)		1.6 (0 - 36)		8% (4)	

When comparing severity of acute pancreatitis and clinical outcome, the patients with severe disease had longer length of stay, more frequently required intensive care and, in such cases, stayed longer at the ICU. There was no correlation between severity of acute pancreatitis and mortality (Table 4).

**TABLE 4:** - Correlation between severity of acute pancreatitis as per the Revised Atlanta classification

**Severity**	**Length of Stay (days)**	**p-value**	**Intensive care required**	**Mortality % (n)**	**p-value**			
			**% (n)**	**p-value**	**days**	**p-value**		
Mild	28.2 (4 - 104)		2% (1)	<0.001 (Y)	0.0 (0 - 1)	<0.001	4.5% (2)	0.138 (Y)
Moderately Severe	51.0 (23 - 58)	0.176	25% (1)		1.8 (0 - 7)		0% (0)	
Severe	45.9 (1 - 91)	0.049	90% (9)		15.2 (0 - 44)		30% (3)	

In the present study the PANC 3 score demonstrated sensibility of 50%, specificity of 100%, accuracy of 91.4%, positive predictive value (PPV) of 100% and negative predictive value (NPV) of 90.6% in prediction of severe acute pancreatitis (Table 5).

**TABLE 5:** - Efficacy of the PANC 3 score in predicting severe acute pancreatitis

	**Sensibility**	**Specificity**	**Accuracy**	**PPV**	**NPV**
PANC 3 +	50%	100%	91.4%	100%	90.6%

p<0.001 (Y); PPV=positive predictive value; NPV=negative predictive value

In an isolated analysis of the PANC 3 score variables, it was observed that pleural effusion presented the best results, including higher sensibility and NPV than the PANC 3 score. Nonetheless, the BMI results only showed a tendency when analyzed separate from the other variables in predicting severe acute pancreatitis (Table 6).

**TABLE 6:** - Efficacy of the isolated PANC 3 score variables in predicting severe acute pancreatitis

	**Sensibility**	**Specificity**	**Accuracy**	**PPV**	**NPV**	**p-value**
BMI	70%	60.4%	62.1%	26.9%	90.6%	0.078
Hematocrit	50%	89.6%	82.8%	50%	89.6%	0.011 (Y)
Pleural effusion	60%	91.7%	86.2%	60%	91.7%	0.001 (Y)

PPV=positive predictive value; NPV=negative predictive value, BMI=body mass index

## DISCUSSION

There are discrepancies in the literature regarding the profile of acute pancreatitis patients, which can be extremely variable^1^^,^ 6 ^,^ 8 ^,^ 13 ^,^ 15 ^,^ 21 ^,^ 26.

It affects more often individuals aged 30 to 60 years^22^; however, it is not limited to this age group, as demonstrated in this study. Due to discrepancies among authors, it seems to have no sex predilection^1^^,^ 6 ^,^ 8 ^,^ 13 ^,^ 15 ^,^ 21 ^,^ 26.

Acute pancreatitis etiology is variable and might be related to some factors, such as age, gender, race, BMI, among others^1^. Approximately 60-70% of cases are caused by biliary lithiasis^2^^,^ 20, and if added to alcohol use, they account for up to 80% of the cases. Roughly 10% of cases have other etiology and in10 to 20% the cause remains unknown^24^^,^ 28. However, current evidence demonstrate that up to one third of idiopathic cases are due to microlithiasis or biliary crystals^20^.

The present study was limited to the general surgery division and this may justify higher frequency of biliary acute pancreatitis. The higher prevalence of women in the sample could also be associated to this fact, since the biliary etiology is more common in females^28^.

Most cases of acute pancreatitis progressed in a mild and self-limited course and the overall mortality was 8-15%^20^. However, nearly 20% of patients progressed as severe cases^9^^,^ 26 ^,^ 28 and usually 10-30% of them died^1^. Mortality rates can be as up as 85%^30^.

As to the PANC 3 score, this study showed better results than that reported in the original article that presented the score; that is, the three positive variables of the score demonstrated a post-test probability of 99% in predicting severe acute pancreatitis^5^. In a similar study carried out by the same division, the findings were also inferior: sensibility of 31.25%, specificity of 100%, accuracy of 83.07%, positive predictive value of 100% and negative predictive value of 81.66%^12^. However when analyzing the PANC 3 score variables isolated, this study found pleural effusion as the most important predictor, differently from the original study, in which hematocrit was a stronger predictor and BMI denoted just a trend^5^.

Comparing the PANC 3 score results obtained in this study with the results of other methods for severe acute pancreatitis prediction, it can be noticed that the PANC 3 score presents similar or superior results, except for its sensibility^15^.

In a further investigation of the severe cases with a negative PANC 3 score, it was observed that out of five patients, two had comorbidities and one who had a mild course suffered a myocardial infarction during hospitalization. In this last case, one could argue if the organ failure that made the patient be classified as severe was not exclusively due to the cardiovascular event, and could be a flaw of the Revised Atlanta classification. Of the patients with previous comorbidities, one had chronic kidney disease and liver failure, both causes of anemia and malnutrition^11^^,^ 14 ^,^ 18 ^,^ 25, which may have led to decreased hematocrit and BMI. The other patient had HIV infection. In accordance with previous studies^7^^,^ 10, it is known that pancreatitis in HIV-positive individuals has a higher risk of progressing to a severe form for different reasons. Furthermore, these patients present more marked anemia and hypoalbuminemia, possibly explaining the lower hematocrit and BMI, and a false negative result in the PANC 3 score. This failure in the methods to predict severity of acute pancreatitis in HIV-infected patients has already been described in previous studies^7^.

This study also reinforces the previously described fact that patients with severe cases stay longer at hospital, and require intensive care more frequently and for a longer period when compared to mild cases^1^^,^ 21. Possibly due to the limited number of patients with severe acute pancreatitis in the sample, no relation was found between severe cases and higher mortality, unlike what was expected^1^^,^ 9 ^,^ 21 ^,^ 28.

The present study also conducted comparisons that have not been analyzed so far, between the PANC 3 score and length of hospital stay, need for and length of intensive care, and mortality. It found that the positive score is related to longer length of stay at intensive care units.

Further studies could argue if the PANC 3 score would have a worse performance in predicting acute pancreatitis in patients with co-morbid conditions.

## CONCLUSION

The PANC 3 score is a useful tool in acute pancreatitis approach, due to its high efficacy, easy application and rapid results, which enables classification of cases and early treatment. 
